# Prevalence and a correlation analysis of discoid meniscus and femoral trochlear dysplasia

**DOI:** 10.1186/s12891-023-06958-x

**Published:** 2023-11-30

**Authors:** Yi Qiao, Xiuyuan Zhang, Chenliang Wu, Caiqi Xu, Zhenkui Sun, Jinzhong Zhao, Song Zhao

**Affiliations:** 1 Department of Sports Medicine, Shanghai Sixth People’s Hospital, Shanghai Jiao Tong University School of Medicine, 600 Yishan Road, Shanghai, 200233 China; 2 Changhai Hospital, Shanghai, China; 3 Department of Medical Imaging and Nuclear Medicine, Shanghai Sixth People’s Hospital, Shanghai Jiao Tong University School of Medicine, 600 Yishan Road, Shanghai, 200233 China

**Keywords:** Discoid meniscus, Femoral trochlear dysplasia, Prevalence, Congenital malformation

## Abstract

**Background:**

Discoid meniscus (DM) and femoral trochlear dysplasia (FTD) are common knee disorders. Both as congenital malformation, whether there is a connection between them is unclear and the research on their prevalence in the general population is inadequate. This study aimed to investigate the prevalence of FTD and DM in the general population through a large sample size, and to explore the relationship between them.

**Study design:**

Retrospective study.

**Methods:**

Patients undergoing knee magnetic resonance imaging (MRI) examinations at our outpatient clinic were screened and 1003 patients were enrolled in DM group with 989 patients in non-DM (NDM) group. The type of DM and FTD was classified with Watanabe classification and Dejour’s classification, respectively. The prevalence of FTD and DM in the general population and the relationship between them were evaluated.

**Results:**

The prevalence of DM and FTD was 10.0% and 14.5%, respectively. The overall percentage of FTD was higher in DM group (P < 0.001). The DM group has a higher percentage of all types of FTD except type D (P < 0.05), and a higher percentage of both low- and high-grade FTD (P < 0.001). There were 633 cases of type I DM and 370 cases of type II DM. The overall percentage of FTD was not significantly different between the two types (P = 0.106). No significant difference was detected for all types of FTD except type B (P < 0.05). The Type I DM group has a significant higher percentage of high-grade FTD than Type II group (P < 0.05).

**Conclusion:**

Patients with a DM are more likely to have FTD regardless of the type of DM, while those with a type I DM are more prone to have a high grade FTD.

## Introduction

Discoid meniscus (DM) and femoral trochlear dysplasia (FTD) are common causes of patellofemoral pain [1–3]. DM is a congenital morphological abnormality, primarily affecting the lateral meniscus with an incidence of 3–5% in the United States and up to 15% in Asian populations [4–6]. Most cases are asymptomatic unless they are torn or unstable [7, 8], thus the true prevalence in general population is unknown and the study about Chinese Han population is insufficient. FTD is also a congenital deformation characterized by a shallow trochlear depth and angle [9–11]. Since there are usually no obvious symptoms in the early stage, the diagnosis of FTD is often overlooked. Though FTD has been demonstrated to be relevant to patellar instability [12–14], the research on its prevalence in the general population is inadequate. In addition, the association between these two congenital malformations remains to be explored. In the clinical practice, patients may be advised to have the DM operated, but in fact they may suffer from symptoms caused by FTD.

Therefore, the purposes of the present study were (1) to investigate the prevalence of FTD and DM in the Chinese Han population through a large sample size, and (2) to explore the relationship between DM and FTD. It was hypothesized that the prevalence of FTD and DM in the general population would be around 15% and that there would be a positive association between the prevalence of DM and FTD.

## Methods

### Study population

This retrospective study was approved by our institution’s review board. From January 2019 to December 2019, 10,076 patients undergoing knee magnetic resonance imaging (MRI) examinations at our outpatient clinic were screened. Patients with a lateral discoid meniscus were included in the DM group. For a sex- and age-matched non-discoid meniscus (NDM) group, patients were selected from the rest subjects and those with a syndromic diagnosis as musculoskeletal manifestations (Marfan syndrome, Ehlers-Danlos syndrome, etc.), cerebral palsy, patellofemoral diseases (except FTD), fractures, ligament injuries or prior knee surgeries were excluded.

### MRI examination and measurement

All patients were examined using the same mode (Siemens, Verio 3.0-T, slice thickness: 3.0 mm) and the images were reviewed by two sports medicine fellowship-trained surgeons to define the type of DM and FTD. For the inconsistency between the results, a second check was performed by the two original reviewers independently and a musculoskeletal radiologist was introduced until the inconsistency resolved.

### Discoid meniscus

**Fig. 1:**
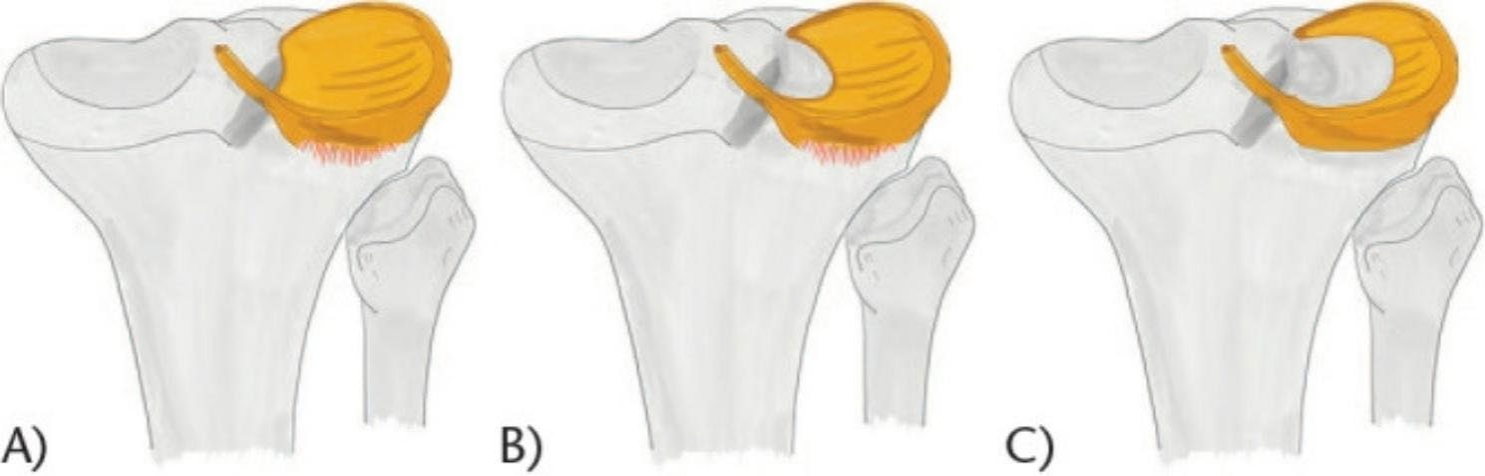
(**A-C**) Watanabe classification for lateral discoid meniscus. (From Saavedra M, Sepúlveda M, Jesús Tuca M, Birrer E. Discoid meniscus: current concepts. EFORT Open Rev. 2020 Aug 1;5(7):371–379; permission under the CC BY-NC 4.0 license)

DM was diagnosed if there were complete and uninterrupted menisci in at least three consecutive sagittal images [15]. The type of DM was classified on the coronal- and sagittal-plane of MRI with Watanabe classification [16] (Fig. 1). Type I has a complete discoid shape, with full coverage of the tibial plateau and normal posterior coronal insertions (Fig. 1A). Type II has a smaller discoid shape, with incomplete coverage of no more than 80% of the tibial plateau (Fig. 1B). Both type I and type II have normal posterior coronal insertions. Type III (the Wrisberg variant) usually has a normal or slightly discoid shape and the posterior coronal fixation is absent, with only the Wrisberg’s ligament maintained (Fig. 1C).

### Femoral trochlear dysplasia

FTD was divided into four types by Dejour’s classification [17, 18]. Type A femoral trochlea has a normal shape, but the trochlea groove is shallow with a groove angle greater than 145°. Type B femoral trochlea is flat or even convex. Type C femoral trochlea is asymmetrical with the lateral femoral condyle being convex and medial femoral condyle being hypoplastic. Type D femoral trochlea has an asymmetrical trochlear facet with a cliff between the facets (cliff sign). Type A was defined as low grade FTD while type B-D comprised high grade FTD [19].

### Statistical analysis

All statistical analyses were conducted with SPSS 26.0. Normality of the data was evaluated with Kolmogorov-Smirnov test. For normally distributed data, ANOVA was conducted, otherwise independent-samples Kruskal-Wallis and Mann-Whitney U test were employed. Chi-square test was used to analyze the relationship between DM and FTD. Fisher’s exact test and Bonferroni correction were adopted when necessary and the level of significance was set at 0.05.

## Results

**Fig. 2:**
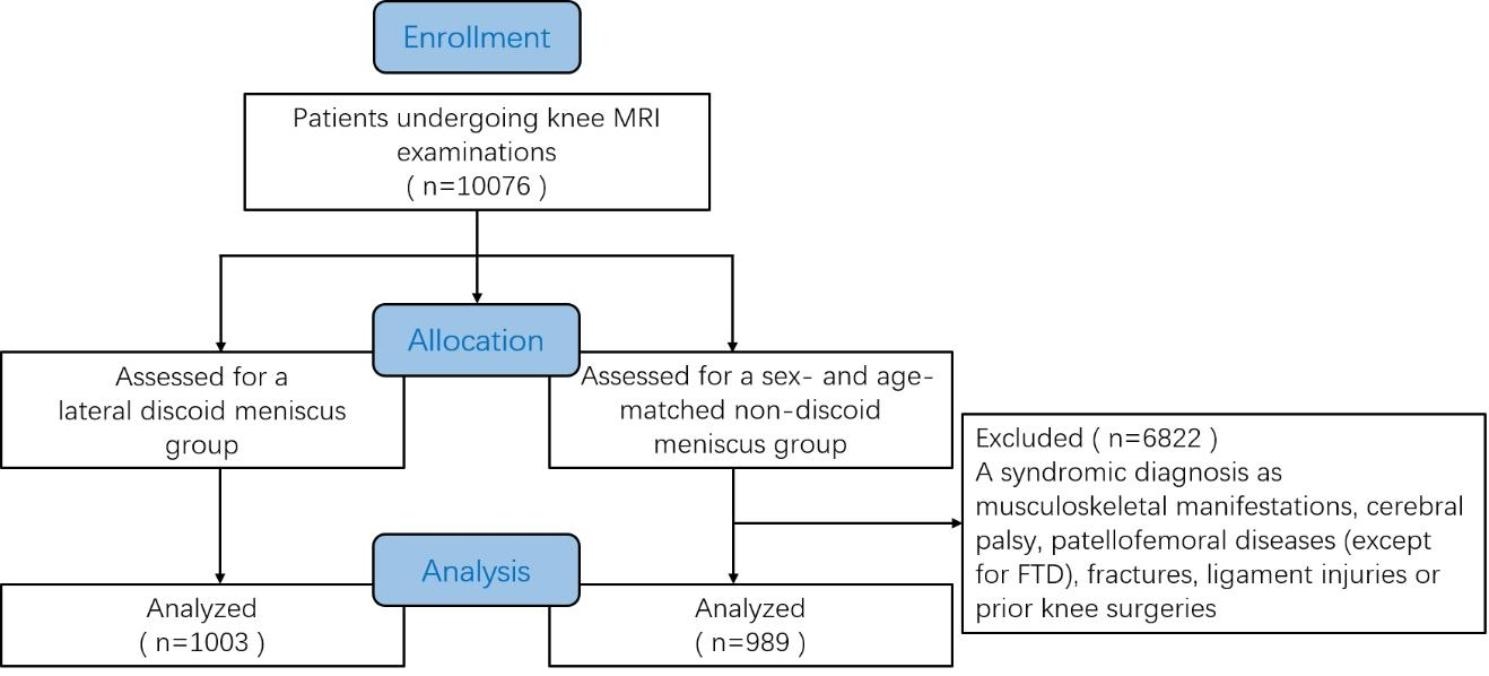
The flow diagram of patient enrollment. MRI, magnetic resonance imaging. FTD, femoral trochlear dysplasia

After screening the 10,076 patients, 1003 patients were included in the DM group. The prevalence of DM in the Chinese Han population was 10.0%. A total of 989 patients were enrolled in the sex- and age-matched NDM group (Fig. 2). The demographic characteristics of the DM and NDM group were shown in Table 1.

**Table 1:** Descriptive Statistics for Patient Demographics

Variables	Non-Discoid Meniscus	Discoid Meniscus
Patients, n	989	1003
Age, median (range)	39 (8–65)	34 (8–64)
Sex		
Female, n (%)	574 (58.0%)	594 (59.2%)
Male, n (%)	415 (42.0%)	409 (40.8%)
Side		
Left, n (%)	491 (49.6%)	503 (50.1%)
Right, n (%)	498 (50.4%)	500 (49.9%)

The number and proportion of each type of FTD in both groups were displayed in Table 2. The overall percentage of FTD was higher in the DM group than that in the NDM group (P < 0.001). For the comparison of type A-D FTD between the two groups, the DM group has a higher percentage of all types except the type D (P < 0.05). For the low grade and high grade FTD, the DM group has a significant higher percentage of both grades than the NDM group (P < 0.001). The NDM group has a significant higher percentage of normal femoral trochlea (P < 0.001). The overall prevalence rate of FTD was 14.5%, with 22.2% in the DM group and 6.6% in the NDM group.

**Table 2:** The number and proportion of different types of FTD in the DM and NDM group

	Non-FTD	Low grade/ A-FTD	B-FTD	C-FTD	D-FTD	High grade/B-D FTD
DM	780 (76.2%)	184 (18.3%)	18 (1.8%)	20 (2.0%)	1 (0.1%)	39 (3.9%)
NDM	924 (93.4%)	61 (6.2%)	3 (0.3%)	1 (0.1%)	0 (0%)	4 (0.4%)

FTD, femoral trochlear dysplasia; DM, discoid meniscus; NDM, non-discoid meniscus

Among the DM group, there were 633 cases of type I DM and 370 cases of type II DM with no case of type III DM. The number and proportion of different types of FTD in each group of DM were provided in Table 3. The overall percentage of FTD was not significantly different between the two groups (P = 0.106). For the comparison of type A-D FTD between the two groups, no significant difference was detected except the type B (P < 0.05). For the low grade and high grade FTD, the Type I DM group has a significant higher percentage of high grade than the Type II DM group (P < 0.05).

**Table 3:** The number and proportion of different types of FTD in each type of DM

	Non-FTD	Low grade/ A-FTD	B-FTD	C-FTD	D-FTD	High grade/B-D FTD
Type I DM	482 (76.2%)	120 (19.0%)	17 (2.7%)	14 (2.2%)	0 (0%)	31 (4.9%)
Type II DM	298 (80.5%)	64 (17.3%)	1 (0.3%)	6 (1.6%)	1 (0.2%)	8 (2.1%)

FTD, femoral trochlear dysplasia; DM, discoid meniscus

## Discussion

The most important finding of the present study was that the prevalence of DM and FTD in the general population was 10.0%, 14.5% respectively. Patients with a DM are more likely to have FTD regardless of the type of DM, while those with a type I DM are more prone to have a high grade FTD.

As a congenital malformation of the meniscus, the prevalence of DM is relatively high in Asian population [20–22]. For Koreans, Kim et al. demonstrated that 14% patients (77 out of 534 arthroscopy cases) in their study were diagnosed with DM [6]. In Japan, Ikeuchi indicated that the prevalence of DM was 16.6% on arthroscopic examination [23]. However, since many asymptomatic DM were found incidentally during surgery [24], the true prevalence in general population is unknown and the study about Chinese Han population is insufficient. The present study noted that the prevalence of DM in the Chinese Han population was about 10.0%, which was similar to the 13% reported by Fukuta et al. who examined the asymptomatic knees of l15 Japanese volunteers with MRI [4]. In line with their study, the present research indicated that asymptomatic DM could occur in any age group.

FTD has been demonstrated to be relevant to various knee disorders such as patellar instability, anterior knee pain and anterior cruciate ligament injury [25]. Though it is common in these populations, the true prevalence of FTD in the general population has been rarely described. DeVries et al. investigated 102 patients’ knees with ultrasound and indicated that the prevalence of FTD in the general population is approximately 10% [12]. In the present study, through an analysis of 1992 patients, the prevalence in the Chinese Han population was about 14.5%. A grasp of such epidemiological data might help clinicians better understand the difference of these disorders between the races and guide patient education.

DM and FTD are relatively common knee diseases, and concurrent cases are often noted [4, 5]. Both as congenital malformation, the current study indicated that there is a correlation between the two disorders, and it might be assumed that there could be a common genetic factor relevant to both disorders. Future researches pertinent to genetic factors might help lead to diagnostic achievements and better understand the development of the malformations. Additionally, the diagnosis of DM is relatively easy and seldom missed, but FTD is often overlooked since there are usually no obvious symptoms in the early stage. The present study demonstrated that patients with a DM are more likely to develop FTD regardless of the type of DM, and those with a type I DM are more prone to have a high grade FTD. Furthermore, on the one hand, clinicians often recommend surgery when detecting a lateral discoid meniscus since DM is relatively easy to diagnose and rarely missed. But in fact, patients may be symptomatic due to FTD, and meniscal surgery could not solve their problem. On the other hand, in patients with DM and FTD, some may have decreased muscle strength after the meniscal surgery [26, 27] and may develop symptoms of patellofemoral instability due to the overlook of FTD and decreased muscle strength. Therefore, for patients with DM, clinicians might consider screening FTD as early detection of trochlear dysplasia could help patients increase their vigilance and better understand their knee conditions to prevent degenerative disorders from being detected until the late stages.

Previous literature has shown differences between men and women, with women having higher rates of trochlear dysplasia [28]. The current study revealed similar findings, with women accounted most for the FTD population. Additionally, the sex-related difference in the DM group was also obvious, suggesting that women are more likely to develop DMs.

This study has several limitations. First, though the sample size was large, this was a single-center retrospective study and the sample was obtained from orthopedic clinics, which may not be sufficiently representative of the general population. Second, patients with certain diseases such as musculoskeletal disorders, fractures or cruciate ligament injuries were excluded. The relationship between DM and FTD in such population could not be analyzed and these exclusions may have artificially lowered the prevalence of DM and FTD. Third, due to the limitation of outpatient conditions, analyses on genetic factors could not be performed. Last, since the sample size of children was relatively small, further analysis to compare the difference between age groups were not powered enough and thus was not conducted.

## Conclusion

Patients with a DM are more likely to have FTD regardless of the type of DM, while those with a type I DM are more prone to have a high grade FTD.

## Data Availability

The datasets used and/or analysed during the current study are available from the corresponding author on reasonable request.
